# Accuracy of Calcium Scoring calculated from contrast-enhanced Coronary Computed Tomography Angiography using a dual-layer spectral CT: A comparison of Calcium Scoring from real and virtual non-contrast data

**DOI:** 10.1371/journal.pone.0208588

**Published:** 2018-12-06

**Authors:** Jonathan Nadjiri, Georgios Kaissis, Felix Meurer, Florian Weis, Karl-Ludwig Laugwitz, Alexandra S. Straeter, Daniela Muenzel, Peter B. Noël, Ernst J. Rummeny, Michael Rasper

**Affiliations:** 1 Department of Diagnostic and Interventional Radiology, Klinikum rechts der Isar, Technical University of Munich, Munich, Germany; 2 Department of Cardiology, Klinikum rechts der Isar, Technical University of Munich, Munich, Germany; 3 Chair of Biomedical Physics & Munich School of BioEngineering, Technical University of Munich, Munich, Germany; Universita degli Studi Magna Graecia di Catanzaro, ITALY

## Abstract

**Purpose:**

Modern non-invasive evaluation of Coronary Artery Disease (CAD) requires non-contrast low dose Computed Tomography (CT) imaging for determination of Calcium Scoring (CACS) and contrast-enhanced imaging for evaluation of vascular stenosis. Several methods for calculation of CACS from contrast-enhanced images have been proposed before. The main principle for that is generation of virtual non-contrast images by iodine subtraction from a contrast-enhanced spectral CT dataset. However, those techniques have some limitations: Dual-Source CT imaging can lead to increased radiation exposure, and switching of the tube voltage (rapid kVp switching) can be associated with slower rotation speed of the gantry and is thus prone to motion artefacts that are especially critical in cardiac imaging. Both techniques cannot simultaneously acquire spectral data. A novel technique to overcome these difficulties is spectral imaging with a dual-layer detector. After absorption of the lower energetic photons in the first layer, the second layer detects a hardened spectrum of the emitted radiation resulting in registration of two different energy spectra at the same time. The objective of the present investigation was to evaluate the accuracy of virtual non-contrast CACS computed from spectral data in comparison to standard non-contrast imaging.

**Methods:**

We consecutively investigated 20 patients referred to Coronary Computed Tomography Angiography (CCTA) with suspicion of CAD using a Dual-Layer spectral CT system (IQon; Philips Healthcare, The Netherlands). CACS was calculated from both, real- and virtual non-contrast images by certified software for medical use. Correlation analyses for real- and virtual non-contrast images and agreement evaluation with Bland-Altman-Plots were performed.

**Results:**

Mean patient age was 57.7 ± 14 years (n = 20). 13 patients (65%) were male. Inter-quartile-range of clinical CACS was 0–448, the mean was 334. Correlation of CACS from real- and virtual non-contrast images was very high (0.94); p < 0.0001. The slope was 2.3 indicating that values from virtual non-contrast images are approximately half of the results obtained from real non-contrast data. Visual analysis of Bland-Altman-Plot shows good accordance of both methods when results from virtual non-contrast data are multiplied by the slope of the logistic regression model (2.3). The acquired power of this results is 0.99.

**Conclusion:**

Determination of Calcium Score from contrast enhanced CCTA using spectral imaging with a dual-layer detector is feasible and shows good agreement with the conventional technique when a proportionality factor is applied. The observed difference between both methods is due to an underestimation of plaque volume, and—to an even greater extend -an underestimation of plaque density with the virtual non-contrast approach. Our data suggest that radiation exposure can be reduced through omitting additional native scans for patients referred to CCTA when using a dual-layer spectral system without the usual limitations of dual energy analysis.

## Introduction

Coronary Artery Disease (CAD) is very common in industrial nations [1]. Early detection and prevention of adverse events is crucial in clinical practice. Consequently, examination numbers are increasing and non-invasive methods like Coronary Computed Tomography Angiography (CCTA) play an increasingly significant role. The accuracy of Computed Tomography (CT)-based imaging for coronary evaluation has been shown to be very high with good additional prognostic value over clinical scores [2–5]. Modern non-invasive evaluation of CAD requires both non-contrast low dose CT imaging for determination of Calcium Scoring (CACS) as a mainly prognostic parameter for cardiovascular disease and adverse events and contrast-enhanced imaging for evaluation of vascular stenosis [6]. Several methods for calculation of CACS from contrast-enhanced images have been previously proposed [7–10]. The main principle underlying those methods is spectral imaging with the possibility to generate virtual non-contrast images by a specific subtraction of iodine from enhanced CT datasets [11]. Spectral CT imaging is becoming more available in the clinical setting allowing for co-registration of two different photon energies [12, 13]. However, the so far proposed techniques for determination of CACS from CCTA have some limitations: Dual-Source imaging and rapid switching of the tube voltage (rapid kVp-switching) cannot simultaneously acquire spectral data [14]. The slight temporal offset between detection of both energy spectra seems critical in cardiac imaging.

A novel technique to overcome these difficulties is spectral CT imaging using a dual-layer detector system. After absorption of the lower energetic photons in the top layer, the bottom layer detects a hardened spectrum of the same emitted radiation resulting in detection of two different energy spectra without temporal offset [15]. Both spectra are detected simultaneously and with the same amount of radiation exposure [13, 14, 16].

The objective of the present investigation was to evaluate the accuracy of CACS from the virtual non-contrast CT images computed from simultaneously acquired spectral data in comparison to standard non-contrast imaging.

## Methods

### Study population

The study was approved by the local ethics committee (Ethikkommission der Fakultät für Medizin der Technischen Universität München). All patients with suspected CAD who underwent CACS and CCTA using spectral CT at our institution were eligible for the study.

### Computed tomography procedure

All examinations were performed on a 64-slice single source dual-layer spectral CT system (IQon; Philips Healthcare, The Netherlands). Firmware on the scanner was 4.7.0. Native and contrast enhanced scans were conducted at 120kVp. Mean tube current for CACS was 364mAs and 549 for CCTA. The native scans were reconstructed with a CB kernel and the CCTA with XCB. In case of heart rates higher than 60 bpm, up to four doses of 5 mg of metoprolol were administered intravenously immediately before scanning. If systolic blood pressure was higher than 100 mmHg, 0.8 mg nitroglycerin was administered sublingually just before scanning to achieve coronary vasodilatation.

Calcium Score was acquired by a non-contrast-enhanced sequential scan.

For timing of the contrast phase Bolus-Tracking was used. The contrast-enhanced scan was obtained using 80 ml of contrast agent (Ultravist 370, Bayer, Bayer AG, Leverkusen, Germany, iodine content 370 mg/ml) at a flow rate of 4–6 ml/s followed by 50 ml of saline chaser bolus.

Each vessel segment with a diameter of more than 1.5 mm was evaluated visually by two experienced radiologists with an experience of more than 500 cardiac CT studies.

The measurements were done by both readers consecutively but independently for each patient at the same console. Before change of reader the evaluation software was reset. Then consent was noted.

### Post-processing

Calcium, Volume and Mass Scores from both, the real and virtual non-contrast images, were evaluated using a commercially available software package certified for medical use (OsiriX MD, OsiriX Foundation, Bernex, Switzerland) with a threshold of 90 in Houndsfield Units(HU) as described before [17–20].

CACS results from OsiriX MD were compared to standard clinical Agatston Calcium Scoring (Philips Intellispace Portal 8.X; Philips Healthcare, The Netherlands).

### Signal-to-noise and contrast-to-noise ratio

The signal-to-noise ratio (SNR) and contrast-to-noise ratio (CNR) were calculated using the methodology described by Szucs-Farkas et al. based on the following equations: SNR = SI ascending aorta / noise and CNR = |(SI ascending aorta—SI myocardium) / noise| [21]. Signal intensity(SI) ascending aorta was the mean of the intravascular density (in HU). Noise was defined as standard deviation (in HU) in the ascending aorta.

### Statistical analysis

Categorical variables are expressed as frequencies and percentages, continuous variables are expressed as mean ± standard deviation or as mean with inter-quartile range. Bland-Altman plots were used for analysis of method agreement. Statistical significance was accepted for two-sided P-values <0.05. After the Bonferroni correction for multiple testing the level of significance for each single test is p = 0.017. The statistical package R version 3.4.0 including the package “BlandAltmanLeh” and “Equivalence” was used for analysis. Post-hoc study power was analysed with GPower.

## Results

### Study population

**Fig 1:**
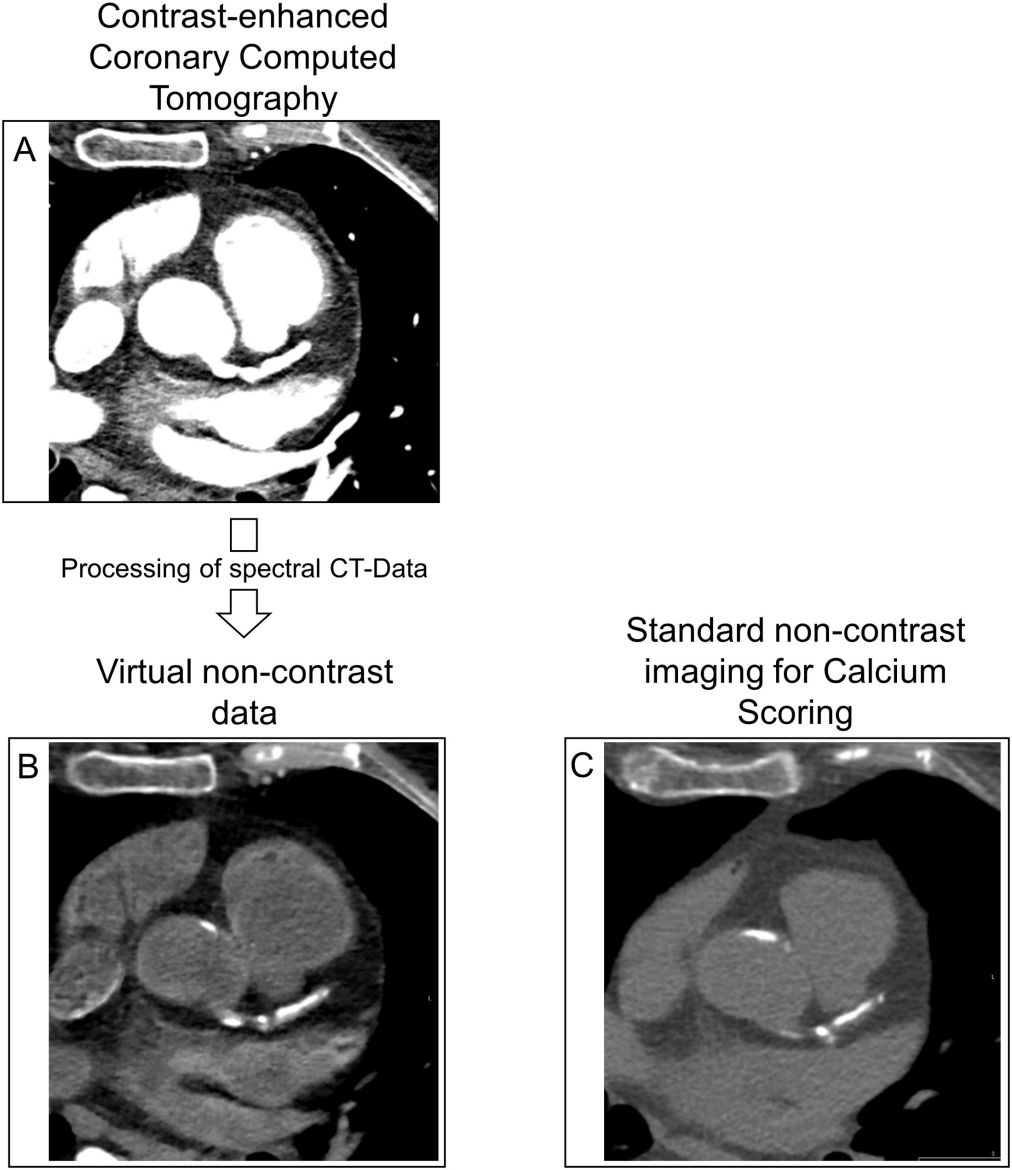
Calcium Scoring from Coronary Computed Tomography Angiography. **A)** Coronary Computed Tomography Angiography (CCTA) after administration of iodine contrast with the dual-layer detector allowing for spectral data acquisition. **B)** Example of a virtual non-contrast image calculated form spectral data set. **C)** Conventional non-contrast imaging for Calcium Scoring in the same patient and slice for comparison.

Between September 2016 and March, 2017 20 consecutive patients with suspected CAD were examined with the dual-layer spectral CT system. The mean age was 57.7 ± 14 years. 13 (65%) were male. The cardiovascular risk factors are shown in Table 1. The mean CACS from real non-contrast was 334 with an inter-quartile-range of 0–448. For virtual non-contrast imaging, mean CACS was 134 and the inter-quartile-range was 0–136. An image example is provided in Fig 1.

**Table 1:** Patients characteristics.

Cardio vascular risk factors	
Age	57.7 ± 14 years
Male gender	13 (65%)
BMI	27.6 ± 5.8 kg/m^2^
Arterial hypertension	11 (55%)
Smoker	current 2 (10%); former 6 (30%)
Diabetes	2 (10.0%)
Hypercholesterolemia	10 (50%)
Positive family history for MI	4 (20%)

BMI = Body Mass Index

### Analysis of CACS results from real non-contrast and virtual non-contrast

**Fig 2:**
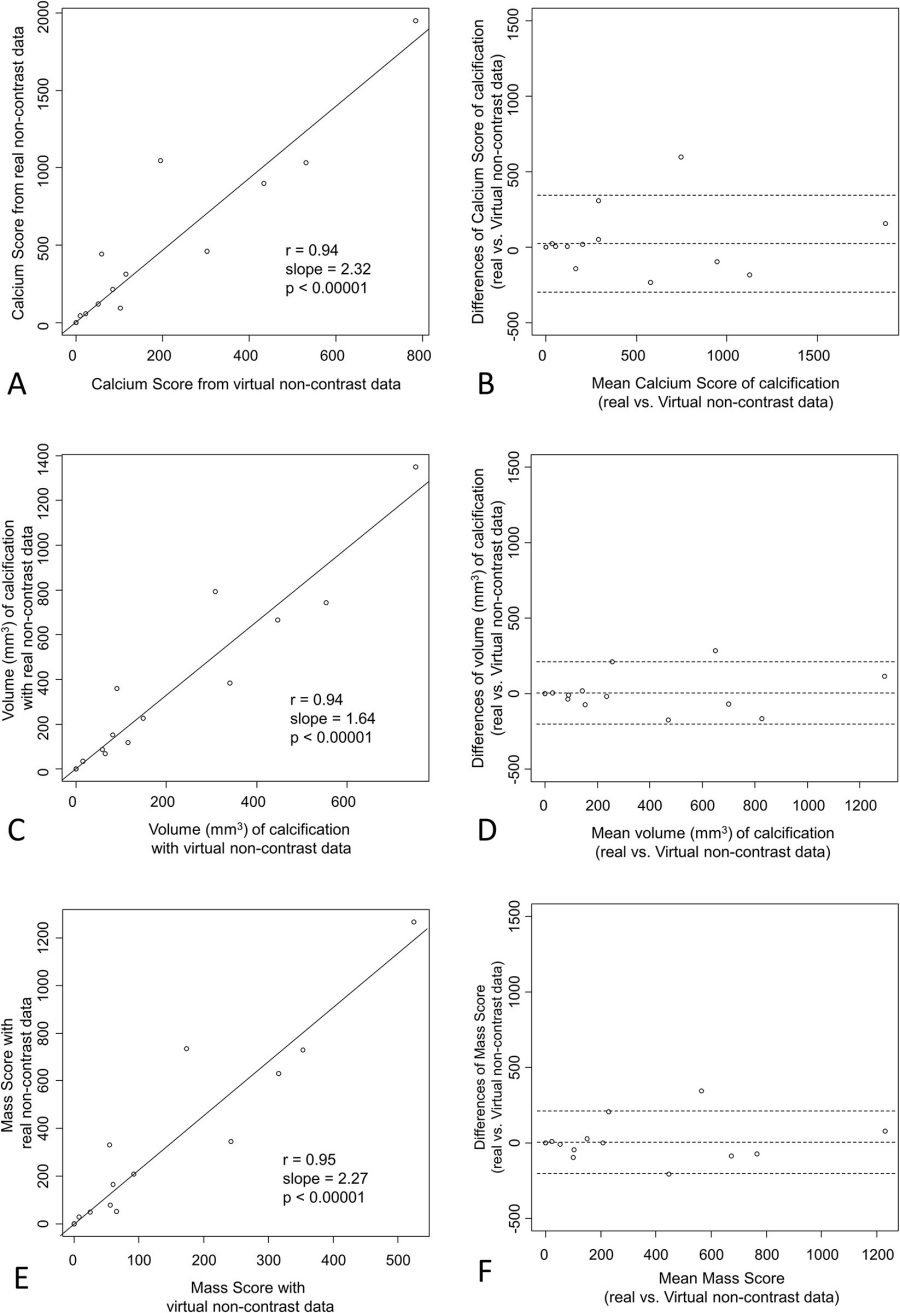
Results of correlation and Bland-Altman analysis. **A** illustrates the correlation between detected Calcium Score using virtual non-contrast and real non-contrast data. Agreement of both methods is shown in **B** with a Bland-Altman plot; the values of the Calcium Score from virtual non-contrast have been corrected by the slope. **C** shows the correlation of the measured volume of calcification between virtual non-contrast and real non-contrast data. The agreement of both methods for determination of the volume of calcification is illustrated in **D** with a Bland-Altman plot; the volume from virtual non-contrast has been corrected by the slope. **E** demonstrates the correlation of the measured Mass Score between virtual non-contrast and real non-contrast data. In **F** agreement of both methods for evaluation of the Mass Score is shown with a Bland-Altman plot; the Mass Score from virtual non-contrast has been corrected by the slope. The outer lines in the Bland-Altman plots visualize 2 standard deviations.

No false positive or false negative calcification was detected using virtual non-contrast images. Correlation of CACS calculated from real- and virtual non-contrast images was very high (0.94); p < 0.0001. The acquired power of this result is 0.99; upper critical r = 0.74. The slope was 2.3 indicating that values from virtual non-contrast images are approximately half of the results from real-non-contrast images (Fig 2A). This slope was used as proportionality constant. Visual analysis of Bland-Altman-Plot (Fig 2B) of CACS shows good accordance between both methods when results from virtual non-contrast data are multiplied by the slope as mentioned above of the logistic regression model (2.3).

### Post hoc analysis

**Fig 3:**
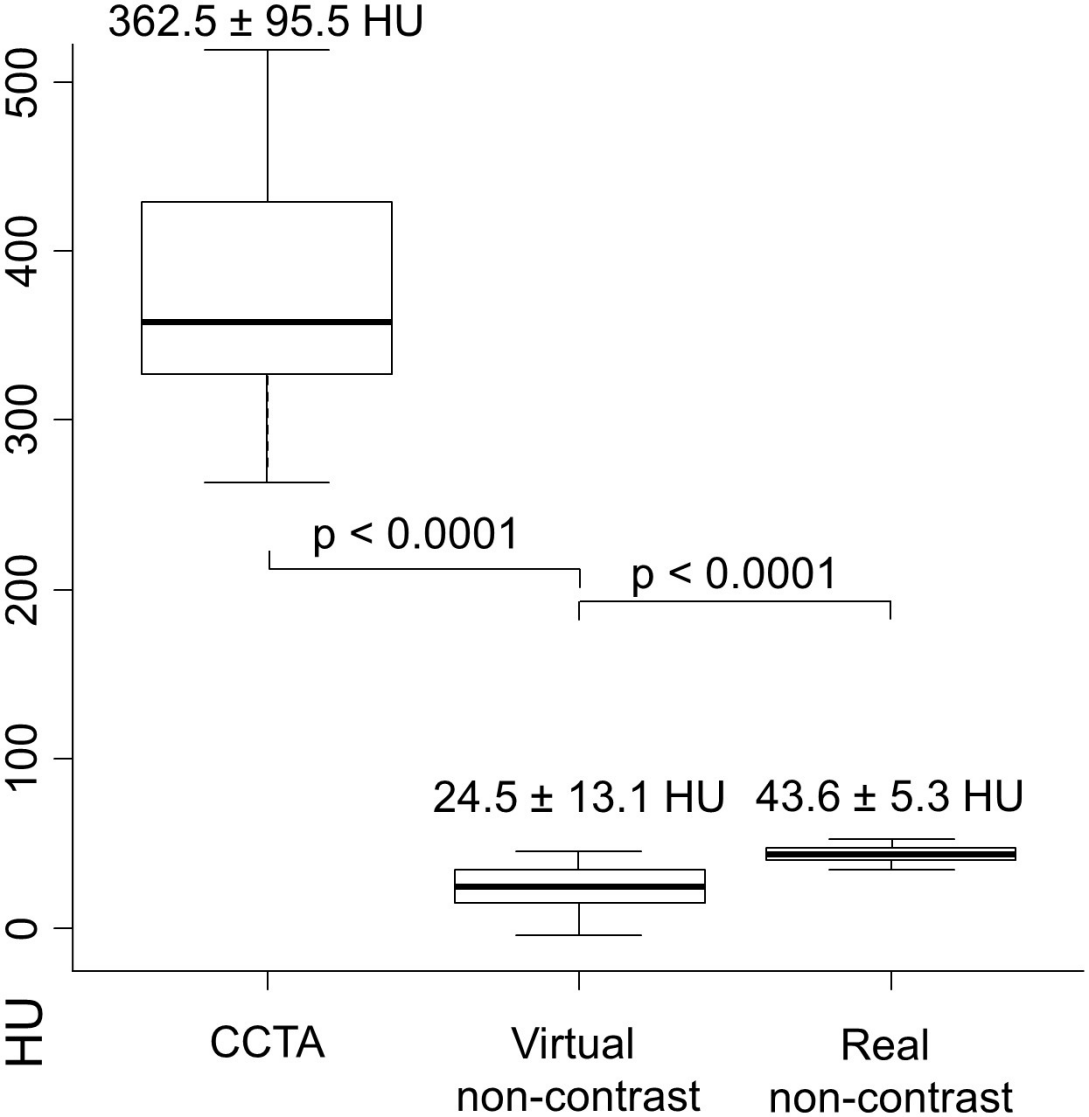
Hounsfield units in the ascending aorta. Boxplots illustrating the statistically significant reduction of HU values in the ascending aorta with the applied virtual non-contrast algorithm measured in axial slices as shown in Fig 1. The mean density in the aorta in the virtual non-contrast images was statistically significantly lower compared to the real non-contrast images. CCTA = Coronary Computed Tomography Angiography.

In general, the application of the virtual non-contrast algorithm led to a statistically significant reduction of measured density in the ascending aorta from 362.5 to 24.5 HU; p < 0.0001 (as shown in Fig 3). The values in the real non-contrast images were statistically significantly higher. The mean SNR for the virtual non-contrast images was 2.6 ± 1.6 and CNR was 1.4 ± 1.6. For real non-contrast images, SNR and CNR was 3.6 ± 1.0 and 1.4 ± 0.5, respectively. SNR and CNR differences between both methods were statistically significant with p = 0.016 and p = 0.0019, respectively.

To evaluate the dependence of CACS results from virtual non-contrast, further correlation analyses have been performed. In the following the influence of volume misregistration, mass misregistration but also the efficiency of the iodine subtraction algorithm on the final CACS results are described.

Correlation of plaque volume between real- and virtual non-contrast images was very high (0.94); p < 0.0001 (Fig 2C). The slope was with 1.64 slower compared to the slope of the CACS between both methods. Good accordance between both methods is shown in the Bland-Altman-Plot (Fig 2D).

There was also very high correlation between the estimated Mass Scores measured on virtual and real non-contrast images (Fig 2D); r = 0.95 with p < 0.0001, slope of the regression line was 2.27 (Fig 2E). In the Bland-Altman-Plot there was good agreement of the methods.

No statistically significant correlation between the difference of CACS calculated from virtual and real non-contrast images and the difference of Hounsfield Units in the aorta in angiography and in virtual non-contrast could be observed; r = 0.1, p = 0.6.

The measured difference in CACS from virtual and real non-contrast images compared to the difference of the Hounsfield Units in the aorta on both non-contrast techniques did not show statistically significant correlation; r = 0.0, p = 0.9.

Further, no relevant correlation between the virtual non-contrast CACS result and the aortic Hounsfield Units in virtual non-contrast images and real non-contrast scans was found.

Additionally, real non-contrast Calcium Scoring from Osirix MD showed very high correlation with clinical standard Agatston (r = 0.99 with p < 0.0001); no statistically significant difference in the results could be observed.

Two-one-sided t-test (TOST) (testing bio-equivalence) results indicate that null hypothesis between statistical difference of corrected CACS from virtual and CACS real non-contrast images can be rejected; p < 0.05. epsilon = 320.

## Discussion

Main findings of this study are: i) there is very high correlation between CACS calculated from a contrast-enhanced spectral CCTA with virtual non-contrast imaging and the standard native technique. With an appropriately chosen proportionality constant (correction coefficient), spectral CT can reliably estimate CACS from a contrast enhanced CCTA. ii) underestimation of calcium score using virtual non-contrast data is a consequence of a slight underestimation of the plaque volume and a larger contribution from the underestimation of plaque density.

Our findings are in line with previously published studies. Schwarz et al. demonstrated high agreement between CACS from a virtual non-contrast data set and the standard evaluation with a dual source CT system [8]. Fuchs et al. used a rapid kVp-switching system for CCTA with a reduced amount of contrast media dose [7]. The group also showed very high correlation (r = 0.96; p < 0.001) of both techniques in principle. However, these methods have theoretically inherent limitations as described before [14]. Both techniques do not acquire the two different energy spectra simultaneously. Depending on protocol and scanner generation, dual source imaging can increase the radiation exposure, although 3^rd^ generation scanner seem to have a more continent dose management [22]. Furthermore, rapid kVp-switching can be limited by reduced rotation times since mAs cannot be switched as fast as kV; moreover, kV-switching can lead to increased radiation exposure [23, 24].

In accordance with previously published data, our results show high effectiveness of the utilized iodine removal algorithm. However, the image quality was inferior to real real non-contrast scans. Further, the iodine removal led to an artificial, but relevant underestimation of density within the aorta. Consecutively, it is to assume that the density of calcified plaques might be underestimated accordingly thus leading to a general underestimation of the uncorrected CACS values. This is supported by the ratio of measured plaque volume comparing the results from real and virtual non-contrast images. The slope of this correlation was with 1.6 closest to 1 of all conducted regression analyses. Still, it is of note that plaque volume is slightly underestimated by virtual non-contrast images as well. In contrast, measurement of Mass Score and CACS showed a strong underestimation of values as their calculations comprise densities; that might be the most important confounder when calculating CACS with virtual non-contrast images. The reasons for underestimation of HU in virtual non-contrast images e.g. in the aorta remain partially unclear and are likely a limitation of the software algorithm.

An improved algorithm to extract virtual non-contrast data from a CCTA might further enhance the reliability of this method. Additionally, a lower threshold for Calcium Scoring for virtual non-contrast imaging might also yield better results as utilization of the same thresholds for both techniques is a possible cause of underestimation of values by the virtual non-contrast approach.

In this study we demonstrate a very high accordance of CACS from real non-contrast and virtual non-contrast imaging. However, there is still a small residual discrepancy. To value this remaining discrepancy, it is of note that interscan variability within 5 minutes at the same scanner has been described before and also notable inter-scan variability for usage of different CT scanners and software has been reported [25, 26]. This underlines the potential of the method proposed in our study to estimate the calcium score with clinical confidence.

It has been discussed before that CACS measured from virtual non-contrast images acquired with dual-energy scanners might be more precise due to a reduction of blooming and beam-hardening [8, 11]; however, the impact of these effects might be different when using a dual-layer detector. Raw data-based beam hardening correction as implemented in the scanner used in our study might yield better results than image based processing [27].

For this reason, a threshold of 90 HU for CACS determination was used in the present investigation. Additionally, this technique has been recommended by several authors for spiral multi-slice CT [28–30] due to reduced noise compared to electron-beam CT where a threshold of 130 HU is recommended. However, results from our study method and Agatston Score matched excellently in a post-hoc test. Translation of our results to other scanners might very likely require establishing of a new correction factor of CACS from real- and virtual non-contrast. This process might even be conducted with a specific phantom and a calibration process. Further studies applying different spectral CTs using just one source of radiation and different software approaches are very worthwhile and mandatory.

### Limitations

This study is a retrospective single centre study. The study population in this study is small. Therefore, the results of this study should be further evaluated with a greater study population. For calculation of scores a threshold of 90 HU was utilized; still, accordance of clinical Agatston Score and the study method was excellent. Although correlation of virtual and real non-contrast CACS was very good after correction, evaluation of the prognostic value of CACS from CCTA with spectral CT is worthwhile but beyond the scope of the recent study.

### Conclusion

Estimation of Calcium Score from contrast enhanced CCTA using spectral imaging with a dual-layer detector is feasible. Radiation exposure can be reduced through omitting native scans for patients referred to CCTA by using dual-layer spectral imaging without the usual limitations of dual energy analysis. Further evaluation of our results in multicentre studies seems worthwhile. An improved algorithm for extraction of virtual non-contrast data from a CCTA might further enhance the reliability of this method.

## Supporting information

S1 Dataset Data set comprising CT values and clinical information. Please see the supporting zipped file: Supporting_DATA.
(ZIP)
